# Structure and Functions of Sidekicks

**DOI:** 10.3389/fnmol.2020.00139

**Published:** 2020-08-25

**Authors:** Masahito Yamagata

**Affiliations:** Department of Molecular and Cellular Biology, Center for Brain Science, Harvard University, Cambridge, MA, United States

**Keywords:** immunoglobulin superfamily, sidekick, Sdk1, Sdk2, adhesion molecule, *Drosophila*, retina, evolution

## Abstract

Many of the immunoglobulin superfamily (IgSF) molecules play pivotal roles in cell communication. The *Sidekick* (*Sdk*) gene, first described in *Drosophila*, encodes the single-pass transmembrane protein, Sdk, which is one of the largest among IgSF membrane proteins. *Sdk* first appeared in multicellular animals during the Precambrian age and later evolved to *Sdk1* and *Sdk2* in vertebrates by gene duplication. In flies, a single *Sdk* is involved in positioning photoreceptor neurons and their axons in the visual system and is responsible for dynamically rearranging cell shapes by strictly populating tricellular adherens junctions in epithelia. In vertebrates, *Sdk1* and *Sdk2* are expressed by unique sets of cell types and distinctively participate in the formation and/or maintenance of neural circuits in the retina, indicating that they are determinants of synaptic specificity. These functions are mediated by specific homophilic binding of their ectodomains and by intracellular association with PDZ scaffold proteins. Recent human genetic studies as well as animal experiments implicate that *Sdk* genes may influence various neurodevelopmental and psychiatric disorders, such as autism spectrum disorders, attention-deficit hyperactivity disorder, addiction, and depression. The gigantic *Sdk1* gene is susceptible to erratic gene rearrangements or mutations in both somatic and germ-line cells, potentially contributing to neurological disorders and some types of cancers. This review summarizes what is known about the structure and roles of Sdks.

## Introduction

The immunoglobulin superfamily (IgSF) is a large group of cell surface or secreted proteins, characterized by the occurrence of a variable number of cognate 70–110 amino acid immunoglobulin (Ig)-like domains, originally noticed in antibodies (Shapiro et al., 2007). Most members of the IgSF have been studied as cell surface receptors, co-receptors, co-effectors, or adhesion molecules. In the immune system, they serve as antigen binding molecules, cytokine receptors, and recognition molecules between distinct classes of immune cells (Barclay, 2003). In the nervous system, they function as neurotrophin receptors (e.g., TrkA) and cell recognition/adhesion molecules (e.g., NCAM, nectins), which play roles in the development and maintenance of nervous tissues and neural circuits (Leshchyns’ka and Sytnyk, 2016; Zinn and Özkan, 2017; Cameron and McAllister, 2018; Sanes and Zipursky, 2020).

Encoding one of the largest IgSFs, the *Sidekick (Sdk)* gene was initially identified in a mutant screen of *Drosophila melanogaster* for defects in eye development. An *Sdk*-null mutant was identified by its rough-eye phenotype, and the gene was suggested to play a role in controlling proper photoreceptor development in the fly eye (Nguyen et al., 1997). The vertebrate ortholog of *Sdk*, *Sidekick-1* (*Sdk1*), was initially identified in a screen for molecular subset markers of retinal ganglion cells (RGCs) in the developing chick retina, and its close homolog, *Sidekick-2* (*Sdk2*), was subsequently identified (Yamagata et al., 2002). By searching the GenBank for *Sdk* homologs in other species, mouse and human *Sdk1* and *Sdk2*, as well as a single *Caenorhabditis elegans (C. elegans) Sdk*, were identified. Mouse *Sdk*s were also cloned using a differential gene expression analysis of HIV-infected versus non-infected kidney cells (Kaufman et al., 2004). *C. elegans Sdk* was later characterized as *RIG-4* (Schwarz et al., 2009). All vertebrates have two *Sdks*, although some species, such as zebrafish, contain extra genes due to gene duplication (Galicia et al., 2018). As discussed later, it appears that non-vertebrate species, including insects and nematodes, have only one *Sdk*.

## Structure

### Domain Organization

**FIGURE 1:**
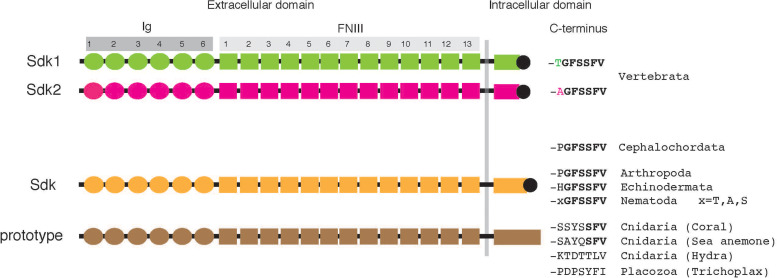
Structure of Sdks. Each Sdk contains a signal sequence, 6 Ig domains (Ig), 13 fibronectin type III (FNIII) domains, a transmembrane domain, and a ∼200–400 amino acid cytoplasmic domain. Vertebrates have two Sdks (also see Table 1). The predicted lengths of typical human Sdk1, human Sdk2, and Drosophila Sdk are 2,213 amino acids, 2,172 amino acids, and 2,168 amino acids, respectively, although each Sdk protein with a cleavable N-terminal signal peptide is translated from spliced mRNA variants and potentially modified by glycosylation.

The predicted vertebrate Sdk1 and Sdk2, as well as fly and worm Sdk proteins, share an identical domain organization. From N to C terminus, each Sdk contains a signal sequence, with 6 Ig domains, 13 fibronectin type III (FNIII) domains, a transmembrane domain, and a ∼200-amino acid cytoplasmic domain (Figure 1). The FNIII domains, originally described in fibronectin, are composed of ∼90 amino acids and have been found in many different proteins, including other extracellular matrix molecules, cell surface adhesion molecules, and receptors. These Sdks possess the unique C-terminal hexapeptide -GFSSFV, which incorporates a tripeptide motif (-SXV) to bind to PDZ domain proteins (Amacher et al., 2020) as discussed below. Vertebrate Sdk1 and Sdk2 are ∼60% identical to each other at the amino acid level, and both are ∼35% identical to *Drosophila* Sdk.

### Evolution of Sdk Structure

It appears that most, if not all, animal phyla have Sdk or Sdk-like molecules (Table 1). All vertebrates have two Sdks: Sdk1 and Sdk2. The sequences of Sdk1 and Sdk2 are conveniently distinguishable by their C-terminal heptapeptide sequences, where Sdk1 and Sdk2 terminate with -**T**GFSSFV and -**A**GFSSFV, respectively (Figure 1 and Table 1). Interestingly, lancelets (amphioxus) have only one Sdk (-PGFSSFV), which is neither Sdk1 nor Sdk2. The genome of this cephalochordate appears to be closer to the genome of the ancestral chordate than those of any other extant organism (Holland et al., 2008). Since cartilaginous fish and teleosts possess Sdk1 and Sdk2, it is likely that *Sdk1* and *Sdk2* were generated by a whole genome duplication event which occurred before the emergence of vertebrates. Supporting this idea, lamprey, a jawless fish, already has two *Sdk* genes. Lamprey is considered to be a link between lancelets and vertebrates (Shimeld and Donoghue, 2012). Lamprey Sdk2 ends with -AGFSSFV, but lamprey Sdk1 contains -SGFSSFV, a non-canonical Sdk1 sequence. In vertebrates, *Sdk1* and *Sdk2* are expressed differentially at the cellular level, often in non-overlapping patterns (see below). The mechanism and contribution of the two Sdks in their body plan is an interesting conjecture.

**TABLE 1:** Sdk1, Sdk2, Sdk, and Sdk prototype.

Species	Common name	Annotation	C-terminal sequence	GenBank Accession #
*Homo sapiens*	Human	Sdk1^1^	-VYTPAGPGARTPLT**GFSSFV**	NP_689957.3
*Mus musculus*	Mouse	Sdk1	-VYTPAGPGARAPLT**GFSSFV**	NP_808547.3
*Monodelphis domestica*	Opossum	Sdk1	-PTGQQAPGSRTPV **GFSSFV**	XP_007498476.1
*Ornithorhynchus anatinus*	Platypus	Sdk1	-PSGQQAPGSRTPV **GFSSFV**	XP_028913331.1
*Gallus gallus*	Chicken	Sdk1	-PTGQPAPGSRTPV **GFSSFV**	NP_989436.2
*Alligator mississippiensis*	Alligator	Sdk1	-PTGQPAPGSRTPV **GFSSFV**	XP_019350208.1
*Rhinatrema bivittatum*	Caecilian	Sdk1	-PTGQQAPGSRTPV **GFSSFV**	XP_029432777.1
*Latimeria chalumnae*	Coelacanth	Sdk1	-PTGQPAPGSRTPV **GFSSFV**	XM_014488585.1
*Danio rerio*	Zebrafish	Sdk1	-PAGQPAPGSRTPV **GFSSFV**	XP_009297968.1
*Amblyraja radiata*	Skate	Sdk1	-PSGQPASGSRTPV **GFSSFV**	XP_032897023.1
*Petromyzon marinus*	Lamprey	Sdk1^2^	-AEGLAGLGPGFTMS**GFSSFV**	XP_032825778.1
*Homo sapiens*	Human	Sdk2	-PPSSLAPGSRAPIA**GFSSFV**	NP_001138424.1
*Mus musculus*	Mouse	Sdk2	-PPSSLAPGSRAPI **GFSSFV**	NP_766388.2
*Monodelphis domestica*	Opossum	Sdk2	-PPSSLAPGSRAPI **GFSSFV**	XP_016286156.1
*Ornithorhynchus anatinus*	Platypus	Sdk2	-PPSSLGPGSRAPI **GFSSFV**	XP_028935753.1
*Gallus gallus*	Chicken	Sdk2	-PPSSLAPGSRAPI **GFSSFV**	NP_989869.2
*Lacerta agilis*	Lizard	Sdk2	-PPSSLAPGSRAPI **GFSSFV**	XP_032994830.1
*Rhinatrema bivittatum*	Caecilian	Sdk2	-PPSSLGPASRAPI **GFSSFV**	XP_029455108.1
*Xenopus tropicalis*	Xenopus	Sdk2	-PPSSLAPAARAPI **GFSSFV**	XP_031750128.1
*Latimeria chalumnae*	Coelacanth	Sdk2	-PPSSLAPGSRAPI **GFSSFV**	XP_014350112.1
*Danio rerio*	Zebrafish	Sdk2	-PPSSLAPGSRAPI **GFSSFV**	XP_009305142.1
*Amblyraja radiata*	Skate	Sdk2	-PASSLAPGSRTPVA**GFSSFV**	XP_032900435.1
*Petromyzon marinus*	Lamprey	Sdk2	-SANGLGPGTRPPVA**GFSSFV**	XP_032822787.1
*Branchiostoma belcheri*	Lancelet (amphioxus)	Sdk	-LANGMAAGSRAPLP**GFSSFV**	XP_019643491.1
*Crassostrea virginica*	Oyster	Sdk	-VIMNNAAGSRAPLP**GFSSFV**	XP_022314291.1
*Octopus bimaculoides*	Octopus	Sdk	-MMVNNTAGSRTPVA**GFSSFV**	XP_029641972.1
*Drosophila melanogaster*	Fruit fly	Sdk	-IIVNNMARSRAPLP**GFSSFV**	NP_001284758.1
*Stegodyphus mimosarum*	Spider	Sdk	-IVMNNMAGSRAPLP**GFSSFV**	KFM81271.1
*Caenorhabditis elegans*	Nematode	Sdk/RIG-4	-GPWANIPATPNLTT**GFSSFV**	NP_501339.2
*Caenorhabditis briggsae*	Nematode	Sdk	-GPWANIPATPNLTA**GFSSFV**	XP_002634371.1
*Oesophagostomum dentatum*	Nodule worm (parasitic nematode)	Sdk	-SSVWQPQPAPNLTS**GFSSFV**	KHJ92754.1
*Strongylocentrotus purpuratus*	Sea urchin	Sdk	-NLAKMQPGSRAPVH**GFSSFV**	XP_030840152.1
*Acanthaster planci*	Starfish	Sdk	-GLAGMPAGSRAPLH**GFSSFV**	XP_022080214.1
*Acropora millepora*	Coral (anthozoan)	Sdk prototype^3^	-YNNDNFSASEPHISSYS**SFV**	XP_029192231.1
*Nematostella vectensis*	Sea anemone (anthozoan)	Sdk prototype	-GATELLDNSEPQISAYQ**SFV**	XP_032221176.1
*Hydra vulgaris*	Hydra (medusozoan)	Sdk prototype	-FNDELKEDEIDGFKTDTTLV	XP_012557393.1
*Trichoplax adhaerens*	Trichoplax	Sdk prototype	-YYHSEQGRVKPGLPDPSYFI	RDD40754.1

*Including arthropods and nematodes, all bilaterian Sdks possess a unique C-terminal hexapeptide -GFSSFV which includes a type I tripeptide motif (-S/T-X-V) for binding to PDZ domain proteins (Bold). The cnidarian and placozoan Sdk-like molecules lack -GFSSFV. Instead, the diversified C-terminal sequences correspond to the type I or type II PDZ-binding motif. Nonetheless, the domain architecture of these Sdk-like proteins is essentially same as that of bilaterian Sdks, making them the prototypes of Sdk. ^1^Sdk1 in other vertebrates: https://www.ncbi.nlm.nih.gov/gene/221935/ortholog/Sdk2 in other vertebrates: https://www.ncbi.nlm.nih.gov/gene/54549/ortholog/^2^*Petromyzon marinus* (sea lamprey) is one of extant agnathan vertebrates that reside at the evolutionary juncture where vertebrates diverged from invertebrates. The C-terminal heptapeptide sequence of *Petromyzon marinus* Sdk1 differs from that of all other vertebrates, although the substitution is relevant (T vs S). At this moment, no *Sdks* are annotated in Urochordata (ascidians). ^3^The domain architecture of non-bilaterian Sdk-like proteins in cnidarians (coral, sea anemone, hydra) and placozoan (trichoplax) is identical to that of bilaterian Sdks (6 Ig domains, 13 FNIII domains, single-pass transmembrane domain, and a cytoplastic domain). However, they do not have -GFSSFV. Currently, no Sdk-like molecules have been annotated in Porifera (sponges) and Ctenophora.*

Besides vertebrates, other bilaterians, including Arthropoda (e.g., insects), Echinodermata (e.g., sea urchin, starfish), and Nematoda (e.g., *C. elegans*) possess one Sdk with -GFSSFV. Each of the non-bilaterians (cnidarians and one placozoa) also has a protein homologous to Sdk. These non-bilaterian Sdk-like proteins have a domain architecture identical to Sdk: 6 Ig and 13 FNIII domains, as well as one transmembrane and cytoplasmic domain. Their cytoplasmic domain is ∼400 amino acids, which is longer than that of vertebrate Sdks, and most strikingly, lacks -GFSSFV. Among cnidarians, Sdk-like proteins in corals and sea anemones end with -SFV, a canonical PDZ-binding motif. However, this -SFV is not present in Sdk-like proteins in *Hydra* and *Trichoplax*. These non-bilaterian animals are a group of the most primitive multicellular animals which appeared in the Precambrian age (Simion et al., 2017), suggesting that these Sdk-like proteins are prototypes of Sdk.

### Ectodomain

*Drosophila* Sdk protein is a homophilic adhesion molecule (Astigarraga et al., 2018). Vertebrate Sdk1 and Sdk2 also show homophilic binding: Sdk1 binds to Sdk1, and Sdk2 binds to Sdk2 (Yamagata et al., 2002; Hayashi et al., 2005; Goodman et al., 2016; Tang et al., 2018). Moreover, neither exhibits heterophilic interactions with other IgSF molecules tested (Yamagata and Sanes, 2008, 2012), although biochemical assays have demonstrated weak cross-binding to other IgSFs under restricted conditions *in vitro* (Visser et al., 2015).

**FIGURE 2:**
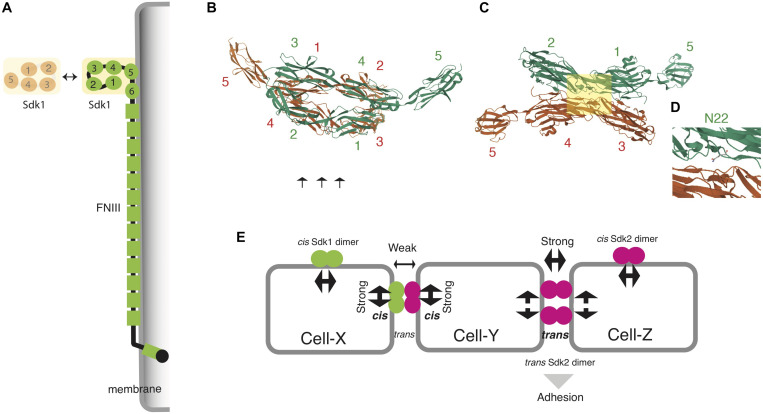
Structural basis of homophilic adhesion in Sdks. **(A)** A horseshoe-like structure of N-terminal Ig domains (Ig1-4) is responsible for homophilic adhesion. Ig5, Ig6, and FNIII domains are associated with plasma membranes (Tang et al., 2018). **(B)** The crystal structure of Sdk1 Ig1-Ig5 homodimers (https://www.rcsb.org/3d-view/5K6W) (Goodman et al., 2016). Four Ig domains of the first molecule (green) faces to those of the second molecule (red) in a back-to-back anti-parallel manner: Ig1 to Ig3, and Ig2 to Ig4. **(C)** The lateral view of **(B)** (arrows in **B**) to display the interactive interface. **(D)** The squared area in **(C)**. The substitution of N22 (Ig1 domain) abolishes the adhesion of *Sdk1*-transfected cells. This residue resides in the interface between Ig1 and Ig3 domains. **(E)** Competition between *cis*- and *trans*- interactions to ensure the homophilic specificity of Sdk-expressing cells (see text). Note that *cis* and *trans* interactions use the same interface as shown in **(B)**.

The structural basis of this homophilic interaction has been revealed by crystal structures and synthetic constructs of Sdk ectodomain regions (Goodman et al., 2016). The four N-terminal Ig domains (Ig1–4) of both Sdk1 and Sdk2 take on a horseshoe-like conformation, like other IgSF proteins (Figures 2A,B), but they interact in a distinct back-to-back anti-parallel manner (Honig and Shapiro, 2020). Amino acid mutations at the interface (especially N22), and Sdk1/Sdk2 chimeric constructs show that this dimer (Ig1-4/Ig1-4 with Ig1:Ig2 and Ig3:Ig4 interfaces) is not only essential for homophilic interaction *in vitro* and cell-cell aggregation (Figures 2C,D) but also forms *cis* Sdk clusters on the cell surface of solitary cells (Figure 2E). Here, only the horseshoe-like structure (Ig1-4) is required for the homophilic binding between two different Sdk molecules (also see Tang et al., 2018). The dimer (Ig1-4/Ig1-4) cannot bind to the second dimer (Ig1-4/Ig1-4) in either *cis* or *trans* because both *cis* and *trans* interactions use the same interface. Thus, to achieve a robust cell–cell adhesion in *trans*, a Sdk molecule on an adjacent cell needs to compete with an Sdk’s *cis* dimer. Interestingly, weak heterophilic binding between Sdk1 and Sdk2 is observed biochemically *in vitro*, although homophilic binding is very strong (Goodman et al., 2016). Here, Sdk1 on Cell-X can bind to Sdk2 on Cell-Y (Figure 2E). However, this heterophilic binding is too weak to pull the Sdk2 away from its cis partner; only another Sdk2 molecule on Cell-Z can do that. Thus, competition between *cis* and *trans* interactions may ensure the homophilic specificity of Sdk-mediated adhesion in the crowded synaptic layers of the central nervous system, where neuronal processes possessing the two Sdks are intermingled.

By contrast, roles of lengthy FNIII domains in Sdk proteins are poorly understood. One possibility is that unknown molecules bind to these domains, although such novel ligands for Sdks have not been reported. An electron microscope analysis of Sdk proteins has demonstrated that the whole ectodomain of Sdk protein has a flexible string-like shape, and that FNIII domains are associated with membranes (Tang et al., 2018). Taken together, the Ig domains of Sdk determine the specificity of *trans* and *cis* interaction, and FNIII domains tighten cell-cell adhesion by closely apposing two cell membranes (Figure 2A).

Sdks have several splicing variants, including a major Sdk1 variant lacking some Ig domains (Kaufman et al., 2004; Yamagata and Sanes, 2019). However, their biological significance has not yet been elucidated.

### Cytoplasmic Domain

**FIGURE 3:**
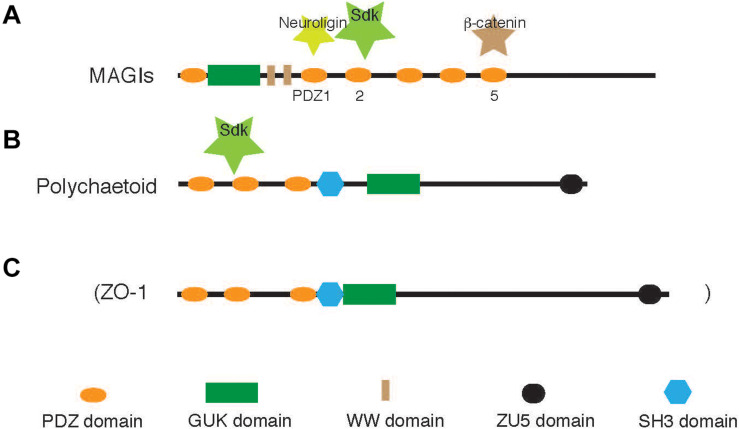
MAGIs and Polychaetoid. The cytoplasmic tail of Sdks binds to two PDZ scaffolding proteins, MAGIs and Polychaetoid, in vertebrates and flies, respectively (Kaufman et al., 2010; Yamagata and Sanes, 2010; Letizia et al., 2019). **(A)** Neuroligin-1, Sdks, and β-catenin bind to the different PDZ domains (see Yamagata and Sanes, 2010). **(B,C)** *Drosophila* Polychaetoid **(B)** is an ortholog of vertebrate ZO-1 **(C)**, a tight junction protein, although its direct interaction with Sdks has not been demonstrated. It is not known which PDZ domains of Polychaetoid bind to Sdk.

Sdks possess a cytoplasmic domain of approximately 200 amino acids, and several clusters of these sequences are conserved across species. Most notably, the C-terminal hexapeptide, -GFSSFV, is conserved in all bilaterian Sdks as discussed earlier. It includes a motif (-SXV) for anchoring to PDZ domain proteins, indicating that it determines the localization of Sdk proteins. It is indeed required for synaptic localization in the retina (Yamagata and Sanes, 2010) and cytoskeletal organization in the kidney podocytes (Kaufman et al., 2010). Using yeast two-hybrid screening, several molecules possessing PDZ domains were identified as robust interactors with this motif (Yamagata and Sanes, 2010), confirming earlier observations (Meyer et al., 2004). Among these interactors, MAGIs, which are one family of PDZ/membrane-associated guanylate kinase (MAGUK) molecules (Figure 3A), colocalize with the Sdk protein in the retina (Yamagata and Sanes, 2010) and kidney podocytes (Kaufman et al., 2010). Thus, Sdk proteins are associated with MAGI proteins *in vivo*. Several lines of evidence suggest that various PDZ-binding motifs show a unique spectrum of binding to distinct PDZ domains in MAGI proteins (e.g., Stiffler et al., 2007). MAGI proteins also directly and indirectly interact with other transmembrane proteins such as neuroligins and cadherins via β-catenin, which are also important components of cell interactions, especially at synapses (Zhu et al., 2016). An intriguing possibility is that MAGI proteins act by orchestrating multiple transmembrane interactions (Yamagata and Sanes, 2010). In addition to MAGIs, it has been shown that *Drosophila* Polychaetoid, another PDZ/MAGUK scaffold protein, is functionally and biochemically associated with the cytoplasmic domain of Sdk (Letizia et al., 2019; Figure 3B). Polychaetoid is a mammalian homolog of ZO-1, which is a major component of tight junctions (Figure 3C). It is interesting to note that these scaffolding proteins can trigger phase separation, which leads to efficient signaling and the high stability of the adhesion apparatus (Su et al., 2016; Canever et al., 2020).

## Functions

### *Sdk in* Drosophila Photoreceptors and Tricellular Adherens Junctions

**FIGURE 4:**
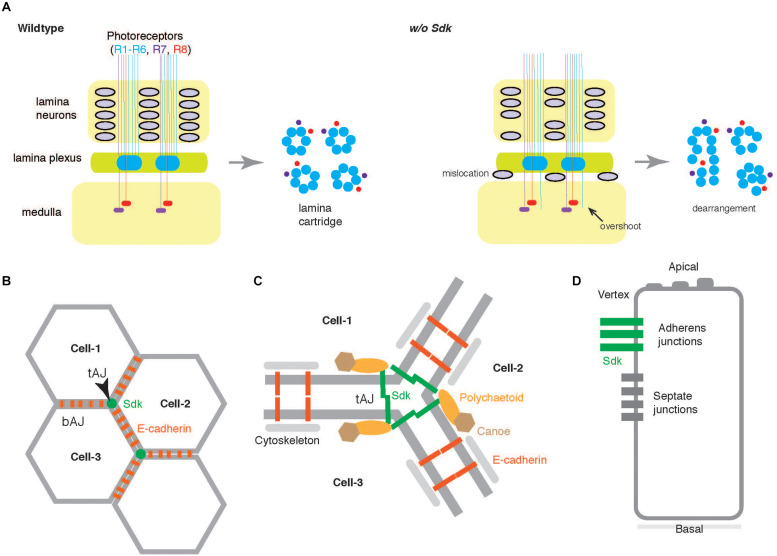
Functions of Sdk in *Drosophila*. **(A)** The compound eyes of the *Drosophila* visual system consist of many ommatidia. Each ommatidium contains eight photoreceptors that project to either lamina (R1–R6) or medulla (R7 and R8). In the lamina plexus, a group of axons from both R1–R6 photoreceptors and lamina neurons make a plexus, which later organized as a lamina cartridge (wildtype, left). In the absence of *Sdk* (*w/o Sdk*, right), the lamina neurons fall off from the packed columns, and the R1–R6 axons occasionally overshoot into the medulla where R7/R8 axons normally terminate (Astigarraga et al., 2018). In the lamina cartridges, the photoreceptor axons are disorganized (transverse section at the right side). **(B)** Epithelial cells build adhesive contacts along their apical-basal axes, both at the bicellular adherens junctions (bAJs) and at the tricellular adherens junctions (tAJs) in *Drosophila*. Sdk is highly concentrated at tAJs which are at the vertex of three mature epithelial cells (Cell-1, Cell-2, and Cell-3) whereas E-cadherin participates in forming bAJ (Finegan et al., 2019; Letizia et al., 2019; Uechi and Kuranaga, 2019) **(B)**. **(C)** At tAJs, the Sdk protein functionally links to Polychaetoid (Figure 3) and Canoe, modulates dynamically E-cadherin by associating with actomyosin cytoskeletons during development, and maintains epithelial sheets. **(D)** Lateral view. In insects, epithelial cells also contact to adjacent cells at septate junctions.

The compound eyes of the *Drosophila* visual system consist of many ommatidia and transmit visual information to the underlying optic lobes via four neuropils:the lamina, medulla, lobula, and lobular plate. Each ommatidium contains eight photoreceptors (R1–R8) which project to either the lamina or medulla (Figure 4A). *Sdk* was initially identified as a gene necessary to control the number and arrangement of cells, including photoreceptors in each ommatidium during *Drosophila* eye development (Nguyen et al., 1997). Further analysis showed that *Sdk* helps to locate lamina neurons, arrange them into columns, and sort photoreceptor axons into lamina cartridges, thereby establishing correct visual motion detection circuits (Astigarraga et al., 2018). For this purpose, *Sdk* is required solely in photoreceptors, but neither in the lamina neurons nor other neurons responsible for motion detection circuits. This mode of action is in contrast to that in the vertebrates where the distinct Sdk mediates homophilic interaction between different cells in *trans* (see below), although *Drosophila* Sdk is a homophilic adhesion molecule (Astigarraga et al., 2018). It raises the possibility that Sdk in flies plays a role in regulating the interaction between photoreceptors and their axons, especially at extending growth cones (Astigarraga et al., 2018). Other models include the expression of heterologous binding partners in the surrounding cells, and/or the release of Sdk fragments from photoreceptors to influence non-cell-autonomously.

Epithelial cells build adhesive contacts along their apical-basal axes, both at bicellular junctions and at tricellular adherens junctions (tAJs) to ensure epithelial integrity, dynamics, and function (Higashi and Miller, 2017; Bosveld et al., 2018) (Figures 4B,C). In a *Drosophila* protein trap project, the GFP-tagged Sdk protein was found to be highly enriched at tAJs (Lye et al., 2014). In an earlier report on the *Sdk*-null mutant (Nguyen et al., 1997), other mysterious phenotypes, such as fused ommatidia, disrupted bristle pattern, and missing pigment cells were also noticed, in addition to photoreceptor abnormalities. In the absence of *Sdk*, disorganization was also seen in several other epithelia such as the epidermis, tracheae, and male genitalia (Finegan et al., 2019; Letizia et al., 2019; Uechi and Kuranaga, 2019). Detailed analyses of these defects revealed that Sdk proteins at tAJs control dynamic junctional rearrangements in developing epithelia. Sdk protein is functionally linked to Polychaetoid and Canoe at tAJs (Letizia et al., 2019) and dynamically modulates the bicellular adhesion molecule, E-cadherin, via actin cytoskeletons (Uechi and Kuranaga, 2019; Figure 4D). Polychaetoid and Canoe correspond to the PDZ/MAGUK protein, ZO-1, and another PDZ protein, afadin, respectively, in vertebrates (Takai and Nakanishi, 2003; Zhu et al., 2016). Sdk can directly bind to Polychaetoid (Letizia et al., 2019). Super-resolution imaging has revealed that Sdk proteins form string-like structures at tAJ vertices (Finegan et al., 2019), indicating that the large Sdk ectodomain is responsible for adopting the structures. It is not clear whether the similar restricted distribution of Sdk proteins contributes to defects of axonal sorting. However, Sdk protein is distributed within small patches associated with axons in the lamina cartridges (Astigarraga et al., 2018), suggesting that the related mechanism may underlie.

### Sdks in Vertebrate Neural Circuits

**FIGURE 5:**
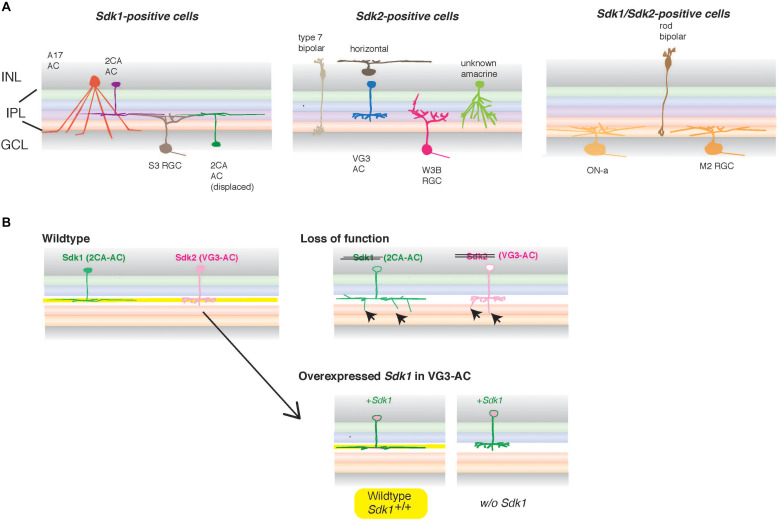
Sdks in vertebrate retinal circuits. **(A)** In the inner plexiform layer (IPL), one of two synaptic layers in the retina, neurites of more than 50 types of interneurons (bipolar and amacrine cells) in the inner nuclear layer (INL) form synapses on dendrites of more than 40 types of retinal ganglion cells (RGCs) in the ganglion cell layer (GCL), thereby assembling the synaptic neuropil consisting of multiple sublaminae. In mice, *Sdk1* and *Sdk2* are expressed by distinct types of retinal neurons (adapted and modified from Yamagata and Sanes, 2019). Some RGC types as well as rod bipolar cells express both *Sdk1* and *Sdk2* (Right panel) (Krishnaswamy et al., 2015; Yamagata and Sanes, 2019). *Sdk2* is expressed by restricted subsets of retinal neurons, including a non-canonical glutamatergic amacrine interneuron (AC) named VGlut3-positive (VG3) ACs and an RGC type called W3B. *Sdk1* is not expressed by these *Sdk2*-positive neurons but expressed by a subset of interneurons (type 2CA cells) and a unique *Sdk1* + RGC. Those *Sdk1*-expressing ACs (2CA) and RGC (S3 RGC) stratify narrowly in the same strata. Similarly, *Sdk2*-expressing amacrine cells (VG3) and W3B-RGC arborize diffusely in the same IPL strata. **(B)** In the absence of *Sdk1*, 2CA-ACs exhibit a reduced sublaminar restriction (arrows) (Yamagata and Sanes, 2019). Similarly, the deletion of *Sdk2* leads to the reduced sublaminar restriction of VG3 arbors as well as dysfunction of this neural circuit with W3B-RGC (Krishnaswamy et al., 2015). Overexpression of *Sdk1* in VG3 cells that normally express *Sdk2* demonstrated that it plays an instructive role in sublaminar targeting in IPL and that it does so only in the presence of Sdk1 in the sublamina (shown in yellow), supporting the “IgSF code” hypothesis for laminar specificity during development. However, this Sdk1-mediated wiring cannot be changed once developed (Yamagata and Sanes, 2019), suggesting that the instructive role of Sdk1 is limited during development.

Vertebrates have two distinct Sdks, which are homophilic. In the developing chick retina, *Sdk1* and *Sdk2* are expressed by non-overlapping subsets of retinal neurons (Yamagata et al., 2002). In mice, a majority of cell types express either *Sdk1* or *Sdk2*, but some cell types express both Sdk1 and Sdk2 (Krishnaswamy et al., 2015; Yamagata and Sanes, 2019; Figure 5A). Likewise, the two proteins are accumulated in the different synaptic layers of the retinal inner plexiform layer (IPL) (Yamagata et al., 2002; Yamagata and Sanes, 2008, 2010, 2012, 2019; Krishnaswamy et al., 2015).

In the IPL, which is one of two retinal synaptic layers, neurites of more than 50 types of interneurons (bipolar and amacrine cells) form synapses on over 40 types of RGC dendrites. This results in the assembly of a synaptic neuropil, consisting of multiple sublaminae (Figure 5A). Functional neural circuits with stereotyped features are formed in each sublamina, since different RGC types selectively respond to specific visual features, such as motion in a specific direction, edges, or color contrasts (Sanes and Masland, 2015). Such laminar specificity in neural circuits is a key feature in many parts of the central nervous system (Sanes and Yamagata, 1999, 2009). A series of experiments using gain-of-function and loss-of-function approaches suggest that both Sdk1 and Sdk2 are required for the restriction of neuronal processes to specific sublaminae within the IPL in chicks and mice (Yamagata et al., 2002; Yamagata and Sanes, 2008, 2019; Krishnaswamy et al., 2015). Their nearest relatives, two Dscams (Dscam and DscamL), and six contactins (Contactin 1–6), are also expressed by neuronal subsets in the chick retina and play relevant roles, formulating the hypothesis that they comprise an “IgSF code” for laminar specificity (Yamagata and Sanes, 2008, 2012).

More specifically, in mice, *Sdk2* is expressed by restricted subsets of retinal neurons, including non-canonical glutamatergic interneurons called Vesicular glutamate transporter-3 (VGlut3)-positive amacrine cells (VG3-ACs), and an RGC type called W3B (Krishnaswamy et al., 2015). W3Bs have the unique property of responding when the timing of small object movement differs from that of the background, but not when they coincide. A line of evidence has suggested that VG3-ACs form synapses on W3B-RGCs; that VG3 input is essential for W3B-RGC function; that *Sdk2* is required for the restriction of VG3-AC and W3B-RGC processes to appropriate sublamina (Figure 5B); and that the number and strength of functional connections between VG3-ACs and W3B-RGCs are specifically diminished in the absence of *Sdk2* (Krishnaswamy et al., 2015). This evidence suggests that Sdk2 has a pivotal role in the formation and/or maintenance of this specific circuit. In mice, *Sdk1* is not expressed by the Sdk2-positive sublamina but is expressed by a subset of interneurons and RGCs that are largely distinct from *Sdk2*-expressing cells. The *Sdk1*-expressing amacrine cells and RGC arborize in the same strata, as well as the neurites of these cells, and all exhibit a reduced sublaminar restriction in the absence of *Sdk1* (Yamagata and Sanes, 2019). Overexpression of *Sdk1* in cells that normally express *Sdk2* demonstrates that *Sdk1* plays an instructive role in sublaminar targeting, and that it does so by a homophilic mechanism (Figure 5B). This evidence further supports the “IgSF code” hypothesis for laminar specificity during development, potentially also in the different parts of the nervous system (e.g., Gu et al., 2015). Moreover, Sdk proteins are found in synaptic sites (Yamagata et al., 2002; Yamagata and Sanes, 2010), indicating that they are involved in specific trans-synaptic interactions.

Thus, in both mice and chicks, two Sdks serve as a part of “IgSF code” for laminar specificity. In mouse retina, the expression and functions of the closest IgSF homologs of Sdks such as Dscams and contactin-5 are similar to those of Sdks: they are expressed in neuronal subsets, and mutations affect the lamination of synaptic layers probably through distinct mechanisms (Fuerst et al., 2008, 2009, 2012; Li et al., 2015; Peng et al., 2017; Simmons et al., 2017). In recent years, other superfamily molecules are implicated for the development of synaptic specificity in various parts of the nervous system, including the vertebrate and invertebrate retina (Yamagata et al., 2003; Sanes and Yamagata, 2009; Shen and Scheiffele, 2010; de Wit and Ghosh, 2016; Sanes and Zipursky, 2020). Sdks play a predominant role in synaptic specificity between RGCs and ACs in the retina. By contrast, in other cell types such as the retinal bipolar cells, distinct adhesion molecules such as type II cadherins play an important role in synaptic specificity (Duan et al., 2018) and constitute a panoply of additional and/or redundant “codes”. In some cases, combinatorial mechanisms could also regulate function of those molecules (Garrett et al., 2018; Yamagata et al., 2018).

The invertebrate and vertebrate retinas share common processing principles but operate through different molecular and cellular mechanisms (Sanes and Zipursky, 2010; Clark and Demb, 2016). Accordingly, mouse Sdk2 and *Drosophila* Sdk share a similar function in visual cue detection but act through distinct cellular mechanisms (Krishnaswamy et al., 2015; Astigarraga et al., 2018). As discussed here, in vertebrates, the Sdk-mediated homophilic adhesion among synaptic partners drives the development of synaptic specificity and function. In Drosophila, *Sdk* is required presynaptically, but not postsynaptically, although it mediates homophilic adhesion molecularly (Astigarraga et al., 2018). Thus, the divergence may include the repurposing of the same mechanism to different anatomical features and the multifunctionality of the same molecule.

## Diseases

### Sdks in Neurodevelopmental and Neurological Disorders

Experimental animal studies have also pinpointed that *Sdk1*-mediated neural circuits may be responsible for addiction and depression. *Sdk1* is upregulated in the nucleus accumbens after chronic cocaine usage in mice (Scobie et al., 2014). In addition, overexpression of *Sdk1* promotes the behavioral effects of cocaine and increases dendritic plasticity in the nucleus accumbens. Sdk1 may also be involved in depression (Bagot et al., 2016; Hultman et al., 2018). *Sdk1* has been identified as a transcript regulated in the brain areas of control mice and those susceptible or resilient to chronic social defeat stress (Bagot et al., 2016). *Sdk1* overexpression in the ventral hippocampus using a herpes virus vector also increases stress vulnerability (Hultman et al., 2018), suggesting that *Sdk1* could be a key factor in understanding stress, such as early life trauma.

In humans, *SDK1* and *SDK2* genes are mapped to 7p22.2 and 17q25.1, respectively. By genome-wide association studies, *SDK1* polymorphism is implicated in autism spectrum disorders (Gai et al., 2012; Connolly et al., 2013; Tsang et al., 2013; Iossifov et al., 2014; Butler et al., 2015; Krishnan et al., 2016; Guo et al., 2019), attention-deficit hyperactivity disorder (Elia et al., 2010; Lima et al., 2016), and motion sickness (Hromatka et al., 2015). In contrast to *SDK1*, *SDK2* has not been noted as a gene linked to many disorders. *SDK2* polymorphism may be related to autism spectrum disorders (Kuwano et al., 2011; Iossifov et al., 2014) and panic disorders (Otowa et al., 2009). Follow-up studies including various transcriptome and connectome analyses are needed to ask if Sdks play roles in these disorders.

In addition to the sequence polymorphisms in *SDK*s, some disease states could be generated because the large *Sdk* genes are unstable and disrupted. During development, DNA double-strand breaks (DSBs) are repaired by non-homologous end joining. Neurons often contain somatic genomic variations caused by this process. *Sdk1* has been identified using an unbiased, high-throughput method, to map genomic regions harboring frequent DSBs in neural stem/progenitor cells (Wei et al., 2016). Most of this repair was observed in long and transcribed genes, including *Sdk1*. This indicates that the *Sdk1* gene is hyperfragile and that this type of recurrent somatic mutation in the *Sdk1* gene *in vivo* could impinge on neurodevelopment and neural functions, as have been discussed for other genes (D’Gama and Walsh, 2018).

In humans, chromosomal anomalies including microduplication and deletion at 7p22 are frequently mapped down to 7p22.1. The 7p22.1 microduplication syndrome is mainly characterized by intellectual disability, speech delay, craniofacial dysmorphisms, and skeletal abnormalities (Ronzoni et al., 2017). However, anomalies in some 7p22.1 syndrome patients extend to 7p22.2, where *SDK1* resides (Cox and Butler, 2015; Ronzoni et al., 2017).

### Sdks in Other Diseases

Kidney disease is among the major causes of mortality in human immunodeficiency virus (HIV)-1-positive patients. *Sdk1* was independently identified in a PCR-coupled subtraction analysis of HIV-1 transgenic versus wild-type immortalized kidney podocytes (Kaufman et al., 2004). *Sdk1*, but not *Sdk2*, was found to be highly upregulated in HIV-1-transgenic podocytes. This suggests a role for Sdk1 in the pathogenesis of glomerular disease in HIV-1-associated nephropathy (Kaufman et al., 2004, 2007). Some SNPs in the human *SDK1* gene are linked to hypertension, although their relationship to renal function has not yet been determined (Tayo et al., 2009; Oguri et al., 2010).

In humans, SDK1 mutations are frequently observed in malignant mesothelioma (Cadby et al., 2013), adrenocortical carcinoma (Juhlin et al., 2015), gastric carcinoma (Rokutan et al., 2016), and lung adenocarcinoma (Mäki-Nevala et al., 2016), raising that possibility that the mutations are related to the etiology of some types of cancers. Other genomic sequences that potentially influence oncogenesis are also seen in the *SDK1* gene (Rezzoug et al., 2016).

Finally, in some prostate cancer patients, gene fusions of *SDK1* to *AMACR* (a-methylacyl-CoA racemase gene) and its transcript have been previously observed (Ren et al., 2012; Zhang et al., 2015). A causal relationship between this *SDK1:AMACR* fusion and prostate cancer progression remains to be clarified.

## Perspective

Sdks are unusually large membrane proteins that have been refractory to structural and biochemical studies. They are often overlooked in molecular screening and systems biology, where the 5’-end of long transcripts is underrepresented. However, recent reports on human *SDK* genes call for further analysis on their pleiotropic roles. *Sdk* is an evolutionarily conserved protein which first appeared in the Precambrian age and later duplicated to generate *Sdk1* and *Sdk2* when vertebrates emerged and evolved. The function of Sdk in primitive multicellular animals is totally unknown. Sdk proteins are concentrated at cell-cell junctions, including at tAJs in *Drosophila*, and at chemical synapses in vertebrates. Inspired by localization of Sdk at tAJs, more studies on vertebrates are required to reveal the precise localization of Sdk proteins at various cell-cell contacts, including synaptic sites, to understand detailed functions of Sdks in diverse neural circuits. Nonetheless, animals without *Sdk* genes are still viable (Nguyen et al., 1997; Yamagata and Sanes, 2019). It is puzzling to consider what kind of selection pressures have enabled *Sdk* to remain in a variety of living and behaving animals.

## Author Contributions

MY wrote the text and created the figures and table.

## Conflict of Interest

The author declares that the research was conducted in the absence of any commercial or financial relationships that could be construed as a potential conflict of interest.
